# Mirror neuron activity is no proof for action understanding

**DOI:** 10.3389/fnhum.2014.00333

**Published:** 2014-05-22

**Authors:** Alina Steinhorst, Joachim Funke

**Affiliations:** Department of Psychology, University of HeidelbergHeidelberg, Germany

**Keywords:** mirror neuron activity, action understanding, action recognition, function of the mirror neuron system, goal coding

## The mirror neuron system and action understanding

Mirror neurons, which have been discovered by single cell recordings in the parieto-frontal areas of the macaque's brain (Rizzolatti et al., 1996), are neurons that discharge in the monkey's brain both when a specific action is observed and when the same action is performed by the monkey himself. In healthy humans, a direct measuring of neural activity is not possible for ethical reasons as the scalp has to be opened for single cell recordings. Still, there is broad evidence from indirect studies that a similar parieto-frontal mirror mechanism also exists in humans (for an overview see Rizzolatti and Sinigaglia, 2008).

In this opinion paper, we will focus on the thesis that action understanding is a function of the mirror neuron system. We will not address intention understanding^1^. According to our opinion, understanding is a process that runs through hermeneutic circles from the “Vorverständnis” (“previous understanding”) to steps of deeper understanding, capturing assigned meaning in its “Bedeutungszusammenhang” (coherence) and recognizing the historical and cultural conditionality of understanding (Dilthey, 1961; Gadamer, 1990). In the following, however, we will focus on the narrow neuroscientific definition of action understanding: the capacity to recognize several movements as belonging to one action. Following Rizzolatti and Sinigaglia (2008), a person “understands” the “action” of a friend moving her arm to an apple if she recognizes this movement to be a grasp toward an apple, if she is able to distinguish it from other movements and if she can use this information to organize appropriate future actions (p. 106). Thus, saying “The person grasps the apple,” she understands the action. This definition equates “understanding” with “recognition,” explaining why sometimes the latter term is chosen (Rizzolatti et al., 1996; Buccino et al., 2004; Iacoboni et al., 2005; Jacob, 2008).

After a reconstruction of the model's developments, we will challenge the claims of the model by Rizzolatti and Sinigaglia (2010). By analyzing the relation between the experimental results and its interpretation, we will conclude that there is no proof that mirror neuron activity leads to action understanding.

## Development of the models linking action understanding to mirror neuron activity

We distinguish between the direct-matching model, the goal mirroring model, and the revised goal mirroring model. This classification is important because the various critiques of the models address different basic assumptions that often are not clearly distinguished from one another. We base our review of the model's developments on an extensive literature research. However, it does not necessarily reflect the classification the advocates would have undertaken.

### Direct-matching model

The direct-matching model was the first to state that “we understand actions when we map the visual representation of the observed action onto our motor representation of the same action” (Rizzolatti et al., 2001: 665; Umiltà et al., 2001; Kohler et al., 2002). Two characteristics of the direct-matching model have provoked criticism:

*First*, it explains human action understanding through “direct matching” of observation and execution. Although not explicitly clarified by the authors, mapping the visual representation onto the motor representation of the *same* action can only be achieved by activation of strictly congruent mirror neurons. The latter constitutes a subclass of mirror neurons that is only activated if the observed and the executed motor act are exactly the same (e.g., grasping with a precision grip). By contrast, broadly congruent mirror neurons code visual and motor acts that belong together without being identical (e.g., grasping with a precision grip or a whole-hand grip). The activation of strictly congruent mirror neurons requires a motor similarity between actor and observer in order to stimulate the exact same subclass of mirror neurons. But as Jacob and Jeannerod (2005) point out, adults describe the movements of geometric stimuli in terms of actions as chasing, attacking, comforting, etc. (Heider and Simmel, 1944). Rhesus monkeys that cannot throw themselves are nevertheless able to understand a throwing action by responding correctly to it (Wood et al., 2007). Thus, “direct matching” as a motor simulation cannot be necessary for action understanding.

*Second*, the direct-matching model does not distinguish between action and goal mirroring. As Csibra (1993) criticizes, in order to understand an action, one has to consider that often there are different means to achieve the same goal. Mirroring can either be a “faithful duplication of observed actions” without taken the goal into account, or it can represent “high-level action understanding” by mirroring the goal of the action without faithfully duplicating the actions (p. 8). Thus, action and goal mirroring cannot be achieved through the same mirror mechanism.

### Goal mirroring model

Discussing the critique of the direct matching model, Rizzolatti and Sinigaglia (2010) now link action understanding not to action, but to goal mirroring: “Through matching the goal of the observed motor act with a motor act that has the same goal, the observer is able to understand what the agent is doing” (p. 269). Unlike in the direct-matching model, this model, that we will call “goal mirroring model,” focuses on the activity of broadly congruent mirror neurons. By coding visual and motor acts that belong together without being identical, broadly congruent mirror neurons seem to mirror the goal underlying different actions. Ferrari et al. (2005) discovered tool-responding mirror neurons in the lateral sector of monkey ventral premotor area F5 that both fire by the observation of an experimenter grasping with a tool and by the monkey's execution of the same grasping act with its hand or mouth. Umiltà et al. (2008) showed that the neuron activity in F5 of two macaque monkeys did not differ when the monkeys used normal or reversed pliers in order to grasp, thus achieving the same goal via the opposite set of movements.

However, as Rizzolatti and Sinigaglia (2010) recognize, action understanding can occur through different mechanisms, making goal mirroring not necessary for action understanding. Humans are able to understand that a dog is barking although there is no activation in parietal-frontal mirror areas (Buccino et al., 2004). As barking does not belong to the human motor repertoire, action understanding cannot exclusively be achieved by mapping the goal of the observed act onto one's own motor repertoire.

### Goal mirroring model (revised)

As a consequence, Rizzolatti and Sinigaglia (2010) modify their concept of action understanding by introducing a gradation in their definition. The “goal mirroring model (revised)” differentiates between the “visual labeling” of an action and its “understanding from the inside.” The authors grant that both types qualify as action understanding, but state that only an “understanding from the inside” is “a real understanding”(p. 270).

Labeling unfamiliar motor acts (“barking,” “saxophone playing” being non saxophone-experts, see Hickok, 2009) is “a mere visual experience.” Motor expertise, that has been shown to elicit stronger mirror neuron activity than non-motor expertise (Calvo-Merino et al., 2005) enables an “understanding from the inside” and thus a “real understanding of the communicative intent and of what saxophone playing really means” (p. 270). Thus, according to this new definition, mirror neuron activity is still conceived as a necessary condition for “real” action understanding.

## The argumentation in the goal-mirroring model (revised)

All experiments cited above (Umiltà et al., 2001, 2008; Kohler et al., 2002; Ferrari et al., 2005) as well as recent experiments (Caggiano et al., 2009, 2012) conduct single cell recordings on monkeys and measure mirror neuron activity. This activity is interpreted as an indicator for action understanding. With reference to previous studies about mirror neuron activity in the human brain, the results are generalized on humans. The goal mirroring model (revised) concludes the primacy of a motor-based action understanding. The argumentation can be broken down into the following 4-step structure:

1. Result: Mirror neuron activity in the monkey's brain is measured.
2. Previous results: Mirror neuron activity also exists in the human brain.
3. Interpretation: Mirror neuron activity is a proof for action understanding “from the inside.”
4. Conclusion: Only through mirror neuron activity, humans understand actions “from the inside.”

In the following section, we will present three points why testing monkey's mirror neuron activity and making statements about human action understanding is incorrect.

### Taking mirror neuron activity as a proof for action understanding (interpretation)

All cited experiments only test the independent variable, mirror neuron activity, by conducting single cell recordings on monkeys. A valid experiment, however, must operationalize the independent *and* the dependent variable. Variations in the independent variable, then, are ideally the causes of variations in the dependent variable. Instead of operationalizing the dependent variable, action understanding, the authors infer from the neural activity in mirror areas that the monkey has understood the action.

*First*, such an inference reduces the monkey on his brain. This form of brain-reading is inaccurate because there is no one-to-one-correspondence between neural areas and their function in cognitive processes. As neural plasticity shows, cognitive processes can be associated with different neural areas. In philosophy of mind, this issue has been discussed 50 years ago: One of the most influential and common objections to the identity theory of mind (which holds that states of the mind are identical to states of the brain) is the argument from “multiple realizability” (Putnam, 1967), criticizing that the same mental state can be implemented by different physical states (see also Place, 1956; Smart, 1959; Block and Fodor, 1972).

*Second*, taking mirror neuron activity as an indicator for action understanding is a tautological argumentation: If mirror neuron activity indicates action understanding, then action understanding has to occur if mirror neuron activity appears. A tautology is not falsifiable and therefore disqualifies as a scientific theory (Popper, 1966). This tautology is fortified in the latest version of the goal mirroring model by Rizzolatti and Sinigaglia (2010). By adding “from the inside” to the term “action understanding,” the latter is by definition linked to mirror neuron activity. The motivation for this appendix remains unclear. In order to show that its purpose is not only to save the own theory, it has to be justified.

### Inferring from mirror neuron activity in the monkey's brain to human action understanding (conclusion)

Even if mirror neuron activity was a proof for the monkey's action understanding, it would not be justified to make statements about *human* action understanding. The narrow definition of action understanding already masks the historical and cultural dimensions of human understanding, but it does not imply an equalization of monkeys' and human action understanding. Such equalization is not proven and not provable: Monkeys cannot be asked about their understanding in an experimental test. Thus, the relation between results, interpretation, and conclusion is methodologically incorrect.

### Stressing the primacy of a motor-based action understanding (conclusion)

Suppose equalizing monkeys' and human action understanding was justified and the results on monkeys could therefore be generalized on humans. Then the experimental results would still not prove the *kind* of statement that Rizzolatti and Sinigaglia (2010) make about human understanding, namely a gradation between “visual labeling” and “understanding from the inside.” It may be intuitively understandable that a person understands a barking dog in another manner than a speaking human individual, as well as expert saxophone players/dancers understand music/dancing in a different way than non-experts. But intuitive plausibility is not a scientific criterion. Following the definition of the authors (section The Mirror Neuron System And Action Understanding), the observer understands the action as long as he recognizes the movements as belonging to one action (“the dog barks,” “the person dances/plays saxophone”). Musical knowledge or the communicative content are part of a broader concept of understanding that has not been tested experimentally. In order to test the primacy of a motor-based action understanding, not only must understanding be tested (see section Taking Mirror Neuron Activity As A Proof For Action Understanding (Interpretation)), but the presumed gradation must be tested, too. Changing definitions in order to account for incompatible results is not a scientific act. It is therefore incorrect to conclude a primacy of a motor-based action understanding.

## Conclusion

In this paper we argue that—contrary to what is postulated in many articles—mirror neuron activity is no proof for action understanding. All cited studies test monkey's mirror neuron activity and make statements about human action understanding. We discussed three points why this is incorrect. *First*, a valid experiment must test both the independent variable (mirror neuron activity) and the dependent variable (action understanding). Taking mirror neuron activity as an indicator for action understanding is a tautological argumentation and an inaccurate reduction of the monkey to his brain. *Second*, even if mirror neuron activity was a proof for action understanding in monkeys, in order to generalize the results on humans, an equalization of monkeys' and human action understanding has to be presumed. Such equalization is not proven and not provable. *Third*, even if mirror neuron activity in monkeys was a proof for action understanding in humans, this would still not prove the primacy of a motor-based action understanding. In support of the goal mirroring model (revised), Rizzolatti and Sinigaglia (2010) do not present experimental results, but a modified yet still tautological definition of action understanding.

We do not conclude that mirror neurons cannot have important social functions. We criticize that the existing experimental results do not support the conclusions. They might be intuitively plausible, but this is not a scientific criterion for a theory to be true. Future studies should measure understanding if they make a statement about it. We conclude that there is no proof that mirror neuron activity leads to action understanding.

## Acknowledgment

We thank Pierre Jacob (Institut Nicod, Paris) for the insightful critical discussions on the function of the mirror neuron system during his visiting professorship at the University of Heidelberg.

### Conflict of interest statement

The authors declare that the research was conducted in the absence of any commercial or financial relationships that could be construed as a potential conflict of interest.

## Footnotes

^1^For a critical discussion of the function of logically related mirror neurons see Borg, 2007. For the broken mirror theory of autism see Cattaneo et al., 2007, for alternative explanations for the social impairment of people with autistic spectrum disorder see Southgate et al., 2009; Frith, 2012.
